# Differentiation of infiltrative cardiomyopathy from hypertrophic cardiomyopathy using high-sensitivity cardiac troponin T: a case-control study

**DOI:** 10.1186/s12872-015-0043-z

**Published:** 2015-06-16

**Authors:** Toru Kubo, Yuichi Baba, Takayoshi Hirota, Katsutoshi Tanioka, Naohito Yamasaki, Shigeo Yamanaka, Tatsuo Iiyama, Naoko Kumagai, Takashi Furuno, Tetsuro Sugiura, Hiroaki Kitaoka

**Affiliations:** Department of Cardiology, Neurology and Aging Science, Kochi Medical School, Oko-cho, Nankoku-shi, 783-8505 Kochi Japan; Department of Laboratory Medicine, Kochi Medical School, Kochi, Japan; Clinical Trial Center, Kochi Medical School, Kochi, Japan

**Keywords:** Infiltrative cardiomyopathy, Hypertrophic cardiomyopathy, High-sensitivity cardiac troponin T

## Abstract

**Background:**

Because infiltrative cardiomyopathy and hypertrophic cardiomyopathy (HCM) share clinical and hemodynamic features of left ventricular (LV) hypertrophy and abnormal diastolic function, it is often difficult to distinguish these entities.

**Methods:**

We investigated the potential role of high-sensitivity cardiac troponin T (hs-cTnT) for differentiation of infiltrative cardiomyopathy from HCM.

**Results:**

The study group consisted of 46 consecutive patients with infiltrative cardiomyopathies or HCM in whom sarcomere protein gene mutations were identified at Kochi Medical School Hospital; of these, there were 11 patients with infiltrative cardiomyopathy (cardiac amyloidosis in 8 patients and Fabry disease in 3 patients) and 35 HCM patients. Serum hs-cTnT level was significantly higher in patients who had infiltrative cardiomyopathy than in those who had HCM (0.083 ± 0.057 ng/ml versus 0.027 ± 0.034 ng/ml, *p* < 0.001), whereas brain natriuretic peptide levels did not differ between the two groups. In two age-matched the 2 cohorts (patients evaluated at > 40 years at age), hs-cTnT level, maximum LV wall thickness, posterior wall thickness, peak early (E) transmitral filling velocity, peak early diastolic (Ea) velocity of tissue Doppler imaging at the lateral corner and E/Ea ratios at both the septal and lateral corners were significantly different between the two groups. As for diagnostic accuracy to differentiate the two groups by using receiver operating characteristic analysis, hs-cTnT was the highest value of area under the curve (0.939) and E/Ea (lateral) was second highest value (0.914).

**Conclusions:**

Serum hs-cTnT is a helpful diagnostic indicator for accurate differentiation between infiltrative cardiomyopathy and HCM.

## Background

**Fig. 1:**
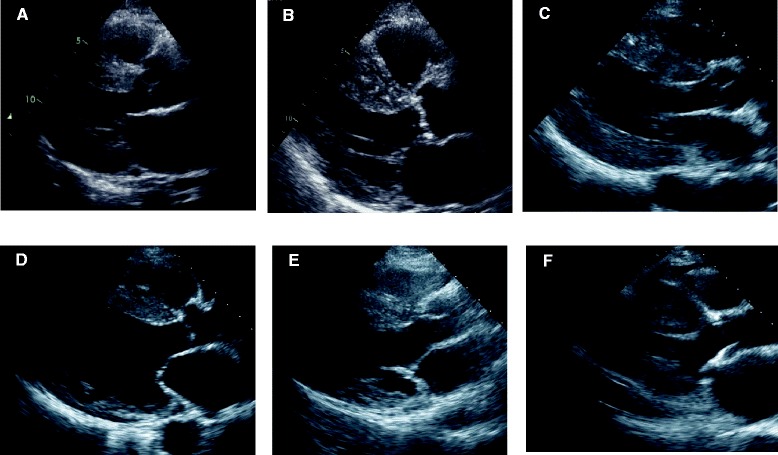
Long-axis two-dimensional echocardiograms (diastole phase) of patients with cardiomyopathies. **a**: a cardiac amyloidosis patient with concentric left ventricular (LV) hypertrophy, **b**: a cardiac amyloidosis patient with asymmetric septal hypertrophy (ASH) at first glance (but actually concentric hypertrophy rather than ASH if moderator band is removed), **c**: a cardiac Fabry patient with concentric LV hypertrophy, **d**: a cardiac Fabry patient with ASH (in a terminal stage patient with Fabry disease, LV systolic dysfunction with localized thinning of the base of the LV posterior wall is seen.), **e**: a hypertrophic cardiomyopathy (HCM) patient with ASH, **f**: a HCM patient with concentric LV hypertrophy at first glance

Infiltrative cardiomyopathies such as cardiac amyloidosis and Fabry disease are difficult to differentiate from hypertrophic cardiomyopathy (HCM) because these cardiomyopathies share clinical and hemodynamic features of left ventricular (LV) hypertrophy and abnormal diastolic function [1–13]. Amyloidosis is a systemic and progressive disease and frequently involves more than one organ. Cardiac involvement in amyloidosis is the most important prognostic factor, and when cardiac amyloidosis is the first or main manifestation of the disease, correct diagnosis is sometimes difficult [1–5]. Fabry disease is a relatively prevalent cause of LV hypertrophy and is associated with significant morbidity and early death due to heart failure or ventricular arrhythmias [6–9, 14]. Since disease-specific enzyme replacement therapy is now available for Fabry disease, correct diagnosis is important [15, 16]. Although cardiac amyloidosis and cardiac involvement in Fabry disease show concentric LV hypertrophy, LV hypertrophy is usually asymmetric and predominantly septal in HCM, and there is often a considerable phenotypic overlap in infiltrative and hypertrophic cardiomyopathies (Fig. 1) [17].

Recently, a new generation of high-sensitivity assays for cardiac troponins has been developed to identify minimal cardiac damage in the setting of acute coronary syndromes [18, 19]. Elevations of cardiac troponin levels in high-sensitivity assays have been reported to be associated with poor outcome in not only ischemic heart disease but also non-ischemic heart failure [20, 21]. Although high-sensitivity troponin levels seem to be different among various cardiomyopathies, there has been no detailed comparison of these biomarkers’ values [22–27]. In the present study, we investigated the potential role of high-sensitivity cardiac troponin T (hs-cTnT) for differentiation of infiltrative cardiomyopathy from HCM.

## Methods

### Subjects

The study group consisted of 46 consecutive patients with infiltrative cardiomyopathies or HCM in whom sarcomere protein gene mutations were identified at Kochi Medical School Hospital; of these, there were 11 patients with infiltrative cardiomyopathy (cardiac amyloidosis in 8 patients and Fabry disease in 3 patients) and 35 HCM patients. In this study, we excluded patients with evidence of coronary artery disease and patients with renal failure (estimated glomerular filtration rate (eGFR) < 30 ml/min per 1.73 m^2^). Informed consent was obtained from all patients or their parents in accordance with the guidelines of the Ethics Committee on Medical Research of Kochi Medical School.

### Clinical evaluation

The diagnosis of amyloidosis was made by biopsy study of an involved organ that demonstrated typical Congo red birefringence when viewed under polarized light. Of the 8 patients with cardiac amyloidosis, 2 had AL amyloidosis and 6 were considered to have senile amyloidosis with transthyretin. The diagnosis of Fabry disease was based on low plasma alfa-galactosidase A activity, family surveys, and tissue confirmation. The diagnosis of HCM was based on echocardiographic demonstration of a hypertrophied, nondilated LV (maximum LV wall thickness ≥ 15 mm) in the absence of systemic hypertension or other cardiac disease (e.g. aortic stenosis) capable of producing clinically evident hypertrophy at some point of clinical course. In this study, all HCM patients with sarcomere gene mutations in whom serum hs-cTnT was measured were enrolled. Sarcomere gene mutations in our current study were S297X, V593fs, V762D, R945fs and R1138C in cardiac myosin-binding protein C gene, R663C and N562K in cardiac beta-myosin heavy chain gene, D46V in cardiac troponin T gene and R162W in cardiac troponin I gene. These were not found in at least 200 chromosomes from healthy Japanese individuals. There were 7 patients with dilated phase of HCM defined as LV systolic dysfunction of global ejection fraction (EF) < 50 %.

Evaluation of patients included medical history, clinical examination, 12-lead electrocardiography, and conventional and Doppler echocardiography. Maximum LV wall thickness was defined as the greatest thickness in any single segment. Left ventricular end-diastolic diameter (LVEDD) and end-systolic diameter (LVESD) were measured from M-mode and 2-D images obtained from parasternal long-axis views. Global EF was determined from apical two- and four-chamber views. Mitral inflow velocities were determined using pulsed-wave Doppler with the sample volume positioned at the tips of the mitral leaflets in the four-chamber view. Peak early (E) and late (A) transmitral filling velocities were measured. Tissue Doppler imaging was performed in the pulse-Doppler mode to allow for a spectral display and recording of mitral annulus velocities at septal and lateral corners. Peak early diastolic (Ea) velocity was measured, and the E/Ea ratio was calculated. LV outflow tract gradient was calculated form continuous-wave Doppler using the simplified Bernoulli equation. LV outflow tract obstruction was defined as the presence of basal LV outflow gradient ≥ 30 mmHg at rest. Right ventricular hypertrophy was defined as thickness of right ventricular free wall > 5 mm.

Peripheral venous blood samples were collected for measurements of serum hs-cTnT and plasma brain natriuretic peptide (BNP) at the same time in clinically stable condition. Blood was taken at a random time mainly in our outpatient clinic. Serum hs-cTnT was measured by Elecsys Troponin T - High Sensitive immunoassay (Roche Diagnostics Ltd., Rotkreuz, Switzerland). The normal range of this troponin marker in an apparently healthy adult population is less than or equal to 0.014 ng/ml (99 percentile). Plasma BNP was measured using an enzyme immunoassay (TOSOH II; TOSOH, Tokyo, Japan).

### Statistical analysis

All data are expressed as mean ± SD or frequency (percentage). Differences between clinical variables of the infiltrative cardiomyopathy group and HCM group were examined with univariate analysis. For analysis of continuous variables, a *t*-test or Wilcoxon rank sum test was used, and a chi-square test or Fisher’s exact test was used for analysis of categorical variables. The diagnostic accuracies of parameters including biomarkers and echocardiographic indices were compared by receiver operating characteristic (ROC) analysis. Age, New York Heart Association functional class, eGFR, E/Ea (lateral), and hs-cTnT were included in a multivariate logistic regression model to identify independent correlations of the differentiation between the two types of cardiomyopathies. Statistical analysis was performed using SPSS (version 14.0 J) statistical software (SPSS Japan Inc., Tokyo).

## Results

### Patients characteristics

**Fig. 2:**
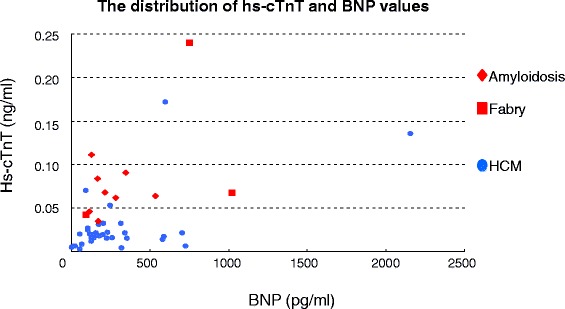
The distribution of hs-cTnT and BNP values. Hs-cTnT: high-sensitivity cardiac troponin T, BNP: brain natriuretic peptide

Clinical characteristics of the patients in the present study are summarized in Table 1. Patients who had infiltrative cardiomyopathy were older than patients who had HCM. Patients with infiltrative cardiomyopathy were more symptomatic than patients with HCM. Figure 2 shows the distribution of hs-cTnT and BNP values in all patients. There was less overlap of hs-cTnT values than BNP values between the two groups. Serum hs-cTnT level was significantly higher in patients who had infiltrative cardiomyopathy than in those who had HCM, while BNP levels did not differ between the two groups (Table 1). Table 2 shows the echocardiographic measurements in the two groups. Maximum LV wall thickness was smaller and posterior wall thickness was greater in patients with infiltrative cardiomyopathy than in those with HCM, although interventricular septal wall thickness was not different. There was no significant difference in the LV or left atrial sizes between the two groups. In Doppler echocardiographic measurements, E wave velocity was higher in patients who had infiltrative cardiomyopathy than in patients who had HCM. Ea at the lateral corner was significantly lower and E/Ea ratios at both the septal and lateral corners were significantly higher in patients who had infiltrative cardiomyopathy than in patients who had HCM.

**Table 1:** Clinical characteristics in 46 patients with cardiomyopathy

	Infiltrative cardiomyopathy (*n* = 11)	HCM (*n* = 35)	p value
Age*, years	68 ± 11	57 ± 16	0.015
Gender: men, n (%)	8 (73 %)	16 (46 %)	0.118
Hs-cTnT*, ng/ml	0.083 ± 0.057	0.027 ± 0.034	<0.001
BNP*, pg/ml	349 ± 341	288 ± 378	0.322
eGFR, ml/min per 1.73 m^2^	61 ± 17	73 ± 21	0.090
NYHA functional class, n (%)			0.071
I	2 (18 %)	20 (57 %)	
II	8 (73 %)	14 (40 %)	
III	1 (9 %)	1 (3 %)	
Hx. of heart failure admission, n (%)	5 (45 %)	6 (17 %)	0.100
Atrial fibrillation, n (%)	1 (9 %)	4 (11 %)	1.00

Data are shown as mean ± SD or number (percent)

A mark of * is the results of Wilcoxon rank sum test

*HCM* hypertrophic cardiomyopathy, *Hs-cTnT* High-sensitivity cardiac troponin T, *BNP* Brain natriuretic peptide, *eGFR* estimated glomerular filtration rate, *NYHA* New York Heart Association functional class

**Table 2:** Echocardiographic findings in 46 patients with cardiomyopathy

	Infiltrative cardiomyopathy (*n* = 11)	HCM (*n* = 35)	p value
Maximum LV wall thickness, mm	17 ± 3	20 ± 4	0.021
Interventricular wall thickness, mm	16 ± 3	16 ± 4	0.628
Posterior wall thickness*, mm	15 ± 3	11 ± 2	<0.001
LV end-diastolic diameter*, mm	44 ± 5	44 ± 6	0.836
LV end-systolic diameter*, mm	32 ± 7	28 ± 7	0.118
Ejection fraction*, %	54 ± 13	62 ± 13	0.104
Left atrial diameter, mm	44 ± 6	43 ± 8	0.723
E*, cm/s	89 ± 23	69 ± 21	0.012
A, cm/s	67 ± 33	64 ± 19	0.704
E/A*	1.7 ± 1.0	1.1 ± 0.5	0.141
Dct*, msec	208 ± 81	200 ± 68	0.857
Ea septal*, cm/s	3.9 ± 1.1	5.2 ± 2.3	0.116
Ea lateral*, cm/s	4.8 ± 1.3	8.1 ± 3.5	<0.001
E/Ea septal	24 ± 7	15 ± 7	<0.001
E/Ea lateral*	20 ± 8	10 ± 4	<0.001
Presence of LVOTO, n (%)	1 (9 %)	7 (20 %)	0.658
Presence of RVH, n (%)	6 (55 %)	18 (51 %)	0.857

Data are shown as mean ± SD or number (percent)

A mark of * is the results of Wilcoxon rank sum test

*HCM* Hypertrophic cardiomyopathy, *LV* Left ventricular, *LVOTO* Left ventricular outflow tract obstruction, *RVH* Right ventricular hypertrophy

### Differentiation of the two groups in patients evaluated at > 40 years of age

**Fig. 3:**
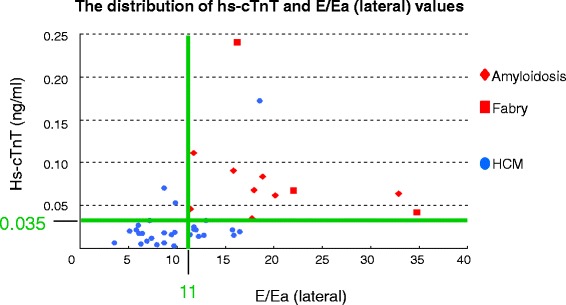
The distribution of hs-cTnT and E/Ea (lateral) values. Hs-cTnT: high-sensitivity cardiac troponin T, E: peak early transmitral filling velocity, Ea (lateral): peak early diastolic velocity of mitral annulus velocities at lateral corner

In clinical practice, phenotypic expression in infiltrative cardiomyopathies is usually after middle-age. We therefore focused on patients evaluated at > 40 years of age in order to clarify the usefulness of hs-cTnT for differentiation of infiltrative cardiomyopathy from HCM. Table 3 shows the clinical characteristics of the two groups of patients evaluated at > 40 years of age. Hs-cTnT level, maximum LV wall thickness, posterior wall thickness, early filling velocity, Ea (lateral) and E/Ea ratios at both the septal and lateral corners were significantly different between the two groups. Area under the curve values in ROC curves are shown in Table 4. Hs-cTnT was highest and E/Ea (lateral) was second highest to differentiate the two groups. The multivariate logistic regression analysis showed that the independent determinants of the differentiation between the two types of cardiomyopathies were hs-cTnT (*p* = 0.047) and E/Ea (lateral) (*p* = 0.028). When the cut-off levels were defined as hs-cTnT of 0.035 ng/ml and E/Ea (lateral) of 11, combined measurements of these parameters resulted in 100 % sensitivity and 95 % specificity for the diagnosis of infiltrative cardiomyopathy (Fig. 3).

**Table 3:** Clinical findings in 41 patients with cardiomyopathy evaluated at > 40 years

	Infiltrative cardiomyopathy (*n* = 11)	HCM (*n* = 30)	p value
Age*, years	68 ± 11	62 ± 11	0.059
Gender: men, n (%)	8 (73 %)	13 (43 %)	0.095
Hs-cTnT*, ng/ml	0.083 ± 0.057	0.025 ± 0.031	<0.001
BNP*, pg/ml	349 ± 301	248 ± 203	0.332
eGFR, ml/min per 1.73 m^2^	61 ± 17	69 ± 19	0.220
NYHA functional class, n (%)			0.124
I	2 (18 %)	16 (53 %)	
II	8 (73 %)	13 (43 %)	
III	1 (9 %)	1 (3 %)	
Hx. of heart failure admission, n (%)	5 (45 %)	6 (20 %)	0.111
Maximum LV wall thickness, mm	17 ± 3	20 ± 4	0.029
Interventricular wall thickness, mm	16 ± 3	16 ± 4	0.562
Posterior wall thickness*, mm	15 ± 3	10 ± 2	<0.001
LV end-diastolic diameter, mm	44 ± 5	45 ± 6	0.877
LV end-systolic diameter*, mm	32 ± 7	28 ± 8	0.161
Ejection fraction, %	54 ± 13	61 ± 13	0.147
Left atrial diameter, mm	44 ± 6	44 ± 9	0.950
E*, cm/s	89 ± 23	69 ± 21	0.019
A, cm/s	67 ± 33	67 ± 19	0.985
E/A*	1.7 ± 1.0	1.1 ± 0.5	0.069
Dct*, msec	208 ± 81	198 ± 70	0.825
Ea septal*, cm/s	3.9 ± 1.1	5.0 ± 2.3	0.226
Ea lateral*, cm/s	4.8 ± 1.3	7.9 ± 3.3	<0.001
E/Ea septal	24 ± 7	16 ± 7	0.002
E/Ea lateral*	20 ± 8	10 ± 4	<0.001
Presence of LVOTO, n (%)	1 (9 %)	5 (17 %)	1.000
Presence of RVH, n (%)	6 (55 %)	14 (47 %)	0.655

Data are shown as mean ± SD or number (percent)

A mark of * is the results of Wilcoxon rank sum test

*HCM* Hypertrophic cardiomyopathy, *Hs-cTnT* High-sensitivity cardiac troponin T, *BNP* Brain natriuretic peptide, *eGFR* estimated glomerular filtration rate, *NYHA*, New York Heart Association functional class, *LV* Left ventricular, *LVOTO* Left ventricular outflow tract obstruction, *RVH* Right ventricular hypertrophy

**Table 4:** Diagnostic accuracy to differentiate the 2 groups. ROC analysis

	AUC value
Hs-cTnT	0.939
E/Ea (lateral)	0.914
Posterior wall thickness	0.906
Ea (lateral)	0.845
E/Ea (septal)	0.806
E	0.742
Maximum LV wall thickness	0.739

*ROC* receiver operating characteristic, *AUC* area under the curve, *Hs-cTnT* high-sensitivity cardiac troponin T, *LV* left ventricular

## Discussion

To the best of knowledge, this is the first report that a new generation of assays, hs-cTnT, is able to differentiate accurately between infiltrative cardiomyopathy, including cardiac amyloidosis and cardiac involvement of Fabry disease, and HCM. Serum hs-cTnT is a helpful diagnostic indicator and should be measured in the evaluation of patients with thickening of the LV wall.

Although the underlying cause is different, infiltrative cardiomyopathy and HCM share clinical and hemodynamic features of LV hypertrophy and abnormal diastolic function [1–13]. It is often difficult to distinguish these entities by routine clinical examinations. Diagnosis of cardiac amyloidosis based on conventional modalities is often only possible once the disease is in a relatively advanced stage [1–5]. In hemodynamic parameters on echocardiography, a restrictive pattern of transmitral flow velocity, which is considered to be characteristic for cardiac amyloidosis, is not always observed in patients with this disease: cardiac amyloidosis can present as an abnormal relaxation pattern, depending on the left atrial or LV end-diastolic pressure. In Fabry disease, cardiac involvement occurs in the majority of patients and is mainly manifested as LV hypertrophy. There have been several reports of Fabry disease sometimes being undiagnosed in HCM cohorts [6–9]. On the other hand, clinical management differs among these cardiomyopathies in terms of prognosis and treatment. Compared with HCM, cardiac amyloidosis and Fabry disease are more progressive and show a poorer prognosis. Furthermore, there are several effective treatments available for these infiltrative cardiomyopathies: high-dose chemotherapy and stem-cell transplantation for AL amyloidosis and enzyme replacement therapy for Fabry disease [15, 16, 28]. To optimize survival in patients with these infiltrative cardiomyopathies, early diagnosis and institution of therapy are essential.

In the present study, we evaluated the value of hs-cTnT to distinguish between infiltrative cardiomyopathies and HCM. Serum hs-cTnT level is a useful marker to differentiate two groups. The mechanisms of myocyte injury and release of cardiac troponins in patients with non-ischemic heart failure or cardiomyopathies remain unresolved. Various reasons have been proposed for high troponin levels, including increased wall stress, myocyte damage from inflammatory cytokines or oxidative stress, altered calcium handling, and coronary microvascular dysfunction [29]. For the hypertrophied myocardium, coronary microvascular dysfunction is considered to be the most plausible mechanism for elevation of cardiac troponins. In fact, microvascular dysfunction has been reported in cardiac amyloidosis, Fabry disease, and HCM [30–32]. Microvascular dysfunction and subsequent ischemia may be important components of the disease progression in patients with cardiac hypertrophy. Although the reason is unclear why hs-cTnT level is higher in patients who had infiltrative cardiomyopathy than in those who had HCM, we speculate that apoptotic or necrotic injury may be induced by the toxic effect of accumulated substances themselves in infiltrative cardiomyopathy.

In the present study, E/Ea at lateral corner (not septal corner) was second highest in area under the curve values in ROC curves to differentiate the two groups. This may result from the findings that amyloid deposition in cardiac amyloidosis is diffuse, whereas LV hypertrophy is usually asymmetric and predominantly septal in HCM.

### Limitations

There are several limitations to be acknowledged in the present study. First, the number of subjects was small and some of the statistical analyses might have been affected. We need to have more data on hs-cTnT levels in various clinical severities in each disease entity. A diagnostic challenge remains in patients with infiltrative cardiomyopathies who have relatively mild abnormalities on echocardiography. Second, due to the retrospective design of the study, it is possible that there is a selection bias, although the study population consisted of consecutive patients with cardiomyopathies. Third, we could not distinguish between cardiac amyloidosis and cardiac involvement of Fabry disease within infiltrative cardiomyopathies by using hs-cTnT measurements. This biomarker has not been fully evaluated in Fabry disease.

## Conclusions

Measurement of serum hs-cTnT enables accurate discrimination between infiltrative cardiomyopathy and HCM. The combination of this biomarker and conventional echocardiographic parameters helps to differentiate these cardiomyopathies.
